# Selective amino acid restriction therapy (SAART): a non-pharmacological strategy against all types of cancer cells

**DOI:** 10.18632/oncoscience.258

**Published:** 2015-11-10

**Authors:** Miguel López-Lázaro

**Affiliations:** ^1^ Department of Pharmacology, Faculty of Pharmacy, University of Seville, Spain

**Keywords:** amino acids, anticancer activity, cancer stem cells, drug resistance, stem cell division theory of cancer

## Abstract

Metastasis will continue to be an incurable disease for most patients until we develop highly selective anticancer therapies. The development of these therapies requires finding and exploiting major differences between cancer cells and normal cells. Although the sum of the many DNA alterations of cancer cells makes up such a major difference, there is currently no way of exploiting these alterations as a whole. Here I propose a non-pharmacological strategy to selectively kill any type of cancer cell, including cancer stem cells, by exploiting their complete set of DNA alterations. It is based on creating challenging environmental conditions that only cells with undamaged DNAs can overcome. Cell survival requires continuous protein synthesis, which in turn requires adequate levels of 20 amino acids (AAs). If we temporarily restrict specific AAs and keep high levels of others whose deficit triggers proteolysis, we will force cells to activate a variety of genetic programs to obtain adequate levels of each of the 20 proteinogenic AAs. Because cancer cells have an extremely altered DNA that has evolved under particular environmental conditions, they may be unable to activate the genetic programs required to adapt to and survive the new environment. Cancer patients may be successfully treated with a protein-free artificial diet in which the levels of specific AAs are manipulated. Practical considerations for testing and implementing this cheap and universal anticancer strategy are discussed.

## INTRODUCTION

Pharmacotherapy is the standard of care for patients with metastasis. When the disease is spread and surgery and radiotherapy are no longer curative, drug therapy becomes the main form of treatment. Pharmacotherapy can prolong patients’ lives and palliate some disease-related symptoms. However, it does not usually cure the disease. The low efficacy of the existing anticancer drugs is reflected in the poor survival rates of patients diagnosed with the most common metastatic cancers. The five-year relative survival rates for patients with distant metastasis are 4% in lung cancer, 28% in prostate cancer, 25% in breast cancer, 13% in colorectal cancer, 16% in melanoma, 12% in renal cancer, 27% in ovarian cancer, 18% in cancers of the uterine corpus, 16% in cancers of the uterine cervix, 6% in bladder cancer, 4% in esophageal cancer, 3% in liver cancer, and 2% in pancreatic cancer [1]. Many patients with metastasis do not overcome the disease despite surviving five years after diagnosis.

Understanding why pharmacotherapy usually fails is important to develop better therapies. When one treats cancer cells with specific concentrations of approved anticancer drugs and examines the cells under the microscope, one generally observes a massacre. All cancer cells die in response to most treatments. However, these same drugs cannot save the lives of cancer patients. The main reason is that these drugs have a limited selectivity towards cancer cells. The consequence of this narrow selectivity is that patients cannot receive the drug doses required to kill all their cancer cells; such doses would also kill their normal body cells and would be lethal. As a poor alternative, they receive the maximum tolerated doses, which are usually insufficient to reach the drug concentrations required to eradicate their cancer cells. The surviving cancer cells continue to proliferate in an uncontrolled way until they eventually lead to a fatal outcome [2].

Pharmacotherapy also fails because some cancer cells are or become resistant to the drugs [3, 4]. The most common reason for resistance is the expression of ATP-binding cassette (ABC) efflux transporters, which eject anticancer drugs from cells. These transporters are expressed in normal stem cells under physiological conditions; these cells have to remain intact for the entire life of an organism and need powerful defense mechanisms against environmental chemical insults. Recent evidence strongly suggests that cancer arises from normal stem cells [5–7]. After accumulating enough DNA alterations, normal stem cells give rise to cancer stem cells (CSCs) [5–7], which keep on expressing ABC transporters [8, 9]. CSCs probably eject the drugs through these transporters and resist therapy. This suggests that even if we developed more selective anticancer drugs, mechanisms that have evolved to protect cells against chemical insults from the environment would continue to act as obstacles to successful treatment of cancer [3].

Cancer pharmacotherapy can also fail because most drugs preferentially target rapidly dividing cells. Resting and slow-proliferating cancer cells, such as CSCs, usually resist therapy. In addition, some resting and slow-proliferating cancer cells are located in poorly vascularized tumor areas. Since the anticancer drugs are delivered to the cells through the blood, tumor cells located in these areas will be exposed to lower drug concentrations than normal cells (which have an adequate blood supply). This factor reduces the already limited selectivity of the existing anticancer drugs and contributes to therapy failure.

Improving the outcome of patients with metastasis requires the development of therapies with a high selectivity towards cancer cells. In addition, these therapies should overcome the drug-resistance mechanisms of these cells. They should also be effective against non-dividing cancer cells and poorly vascularized tumor cells. Here I describe a therapeutic strategy that may fulfill all these requirements.

### Searching for selective anticancer therapies

The main limitation of cancer pharmacotherapy is its low selectivity towards cancer cells. With the discovery of CSCs, it has often been assumed that the main limitation of the existing treatments is their inability to kill CSCs [10]. Evidence has accumulated that pharmacotherapy is ineffective at killing CSCs. However, this does not mean that the existing drugs can selectively kill the rest of cancer cells. As discussed elsewhere, the problem for most cancers is not that a few cancer cells survive treatment, but that only a few cancer cells die in response to treatment [11]. Successful cancer therapy requires the development of therapies with a high selectivity towards all types of cancer cells.

The basis for developing selective anticancer therapies is similar to that for developing selective anti-infective treatments. The aim is to eliminate the infectious agent or the cancer cells without harming the patient too much. The way is to find major and exploitable differences between our cells and the infectious agent, or between our normal cells and the cancer cells. For example, unlike human cells, most bacteria have a cell wall. This major difference can be exploited by inhibiting cell wall synthesis with antibiotics such as penicillins. Because antibiotics can kill bacteria without significantly affecting human cells, they usually save the lives of people with bacterial infections. Saving the lives of patients with metastatic tumors requires finding major and exploitable differences between cancer cells and normal cells.

There exists a major difference between normal cells and all types of cancer cells: unlike normal cells, cancer cells have an extremely altered DNA. As explained elsewhere [12], if one looks at most tumor cells, it looks like someone set off a bomb in the nucleus. There are big pieces of chromosomes hooked together and gains and losses of whole chromosomes in most tumor cells [12, 13]. The karyotype of some tumor cells is strikingly different from that of normal cells; for example, some studies have reported malignant cells with over 100 chromosomes (http://cgap.nci.nih.gov/Chromosomes/Mitelman). Within chromosomes, thousands of DNA mutations and epigenetic alterations are present in most tumors [14–16]. There are usually between 1,000 and 10,000 mutations in the genomes of most adult cancers, including breast and colorectal cancers. Some cancers carry fewer mutations (e.g., testicular germ cell tumors and some leukemias). Others, such as lung cancers and melanomas, have many more mutations (occasionally more than 100,000) [14]. It is actually surprising that cells with so many DNA alterations are able to survive.

Current therapies do not fully exploit this major difference between cancer cells and normal cells. The new drugs are usually designed to target single DNA defects of malignant cells. For example, cancer cells commonly have mutations in genes encoding particular protein kinases. Because these proteins play an important role in cancer cell proliferation, many of the drugs recently approved for cancer therapy have been designed to inhibit specific kinases. However, exploiting minor differences between cancer cells and normal cells usually leads to minor improvements in patient survival. It has been estimated that the recent approval of 71 anticancer drugs has only led to a median overall survival benefit of 2.1 months, balanced against an estimated 10,000 dollars per month on therapy at a cost of 2.7 million dollars per life year saved [17–20]. Current trends suggest that successful therapy of a particular cancer may require finding drugs for each of the driving mutations of that cancer. Given the complexity and variability of the cancer genome, the clinical benefit of this strategy may be limited [21,22].

The key to developing highly selective anticancer therapies probably lies on finding a way to exploit the complete set of DNA alterations of cancer cells. Here I discuss that this can be achieved by creating a challenging cellular environment that only cells with undamaged DNAs can overcome. Normal cells would use their intact DNA to activate genetic and epigenetic programs to adapt to and survive the new conditions. Cancer cells, however, may be unable to survive in the new environment. The activation of these adaptation programs may require the expression of genes that, in cancer cells, may be lost, mutated or silenced. Some of these genes may be in chromosomes or pieces of chromosomes that were lost during carcinogenesis. Others may be mutated and non-functional. In addition, the activation of a genetic or epigenetic program may require changes in other programs that cancer cells may need to keep unchanged for survival.

We can create a lethal environment for cancer cells without drugs. Because surgery and radiation therapy cannot eliminate non-localized tumor cells, we often assume that drug therapy is the only possible way to successfully treat patients with metastasis. By entering the bloodstream, a drug can potentially reach and kill any non-localized cancer cell. Although we can kill cancer cells by administering a cytotoxic agent, we can also kill them by restricting something they need to survive. The result seems to be the same; however, targeting cancer cells without drugs may overcome many drug-resistance mechanisms of cancer cells (e.g., there are no drugs to pump out of the cells through ABC transporters). In addition, the location of cancer cells in poorly vascularized tumor areas may not compromise the efficacy of a restriction therapy.

### Selective killing of cancer cells by amino acid restriction

Cell survival requires protein synthesis. Proteins are continuously degraded and replaced with new ones to ensure a constant supply of functional proteins. The rate of turnover varies widely from protein to protein; the median has been estimated to be 0.5-35 hours in dividing cells and approximately 43 hours in non-dividing cells [23–25]. Protein synthesis in humans requires adequate levels of the 20 canonical amino acids (AAs). An inadequate supply of just one of them for long enough will jeopardize protein synthesis and will result in cell death. Many proteinogenic AAs are also necessary for other cellular processes. All cancer cells, including CSCs, non-dividing cancer cells, or any type of resistant cancer cell, will die if they do not obtain adequate levels of any proteinogenic AA.

**Figure 1:**
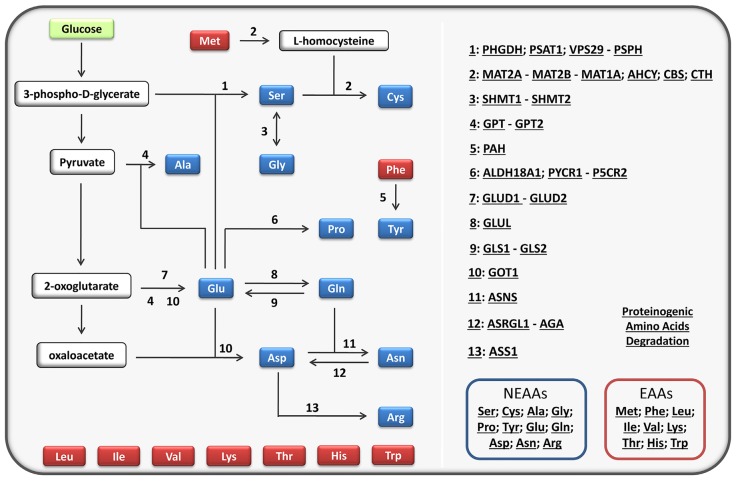
Proteinogenic amino acids The left part of the figure shows the proteinogenic amino acids and the main biosynthetic pathways for the non-essential amino acids (NEAAs). Selenocysteine [63] is not included for simplicity. The NEAAs are represented in blue and the essential amino acids (EAAs) in red. The right part of the figure provides links to the biosynthetic pathways, enzymes and amino acids. It also provides a link to their degradation pathways. The links provide useful information about the chromosome location of the genes coding for the enzymes, the tissue distribution of the enzymes, and the reactions known to produce and consume each amino acid. Most information was taken from HumanCyc: Encyclopedia of Human Genes and Metabolism (http://humancyc.org/). The interactive figure can be found in the Supplementary Figure 1. Ser: L-serine; Cys: L-cysteine, Ala: L-alanine; Gly: glycine; Pro: L-proline; Glu: L-glutamate; Gln: L-glutamine; Asp: L-aspartate; Asn; L-asparagine; Arg; L-arginine; Met: L-methionine; Phe: L-phenylalanine; Leu: L-leucine; Ile: L-isoleucine; Val: L-valine; Lys: L-lysine; Thr: L-threonine; His: L-histidine; Trp: L-tryptophane.

AA restriction can result in selective killing of cancer cells. Human cells cannot synthesize nine of the 20 proteinogenic AAs; these nine AAs are referred to as essential AAs (EAAs) and need to be taken from the diet. The rest, called non-essential AAs (NEAAs), can be synthesized from glucose and from some essential and non-essential AAs. The biosynthesis of NEAAs requires a variety of enzymes that catalyze several reactions and pathways (Figure 1). Some genes encoding these enzymes may not be functional in cancer cells; they may be mutated, silenced or located in lost chromosomes. However, since dietary proteins provide each of the 20 AAs required for protein synthesis, these DNA alterations would not jeopardize the survival of cancer cells. This could change with a protein-free artificial diet in which the levels of particular NEAAs are temporarily restricted. Cancer cells with defects in the synthesis of a specific AA would not survive restriction of this AA, while normal cells would. This is supported by the clinical use of the anticancer drug asparaginase. It has been known for several decades that some leukemic cells have deficient expression of the enzyme asparagine synthase (ASNS), which results in deficient synthesis of the NEAA asparagine. Because normal cells can properly synthesize asparagine, its hydrolysis by asparaginase results in selective killing of leukemic cells [26]. Following asparagine restriction by asparaginase, normal cells synthesize this NEAA and survive, while leukemic cells do not synthesize it and die.

Amino acid restriction can also be lethal for cancer cells without mutations in genes involved in the synthesis of NEAAs. Carcinogenesis is an evolution process in which normal cells acquire multiple DNA alterations. However, not all of them provide a survival benefit. Since many DNA alterations are incompatible with cell survival under specific environmental conditions, cells can only acquire those alterations that allow them to survive in the existing environment. It is important to realize that carcinogenesis takes place under environments in which the levels and ratios of the 20 proteinogenic AAs remain relatively constant. The main reason is that virtually all food proteins contain each of the 20 proteinogenic AAs (gelatin lacks tryptophan), and a standard diet usually provides AAs at relatively constant ratios. However, we can alter the environment under which cancer cells have evolved with a protein-free artificial diet in which the levels of particular AAs are manipulated. This new environment may cause their death, because the DNA alterations that provide a survival benefit under specific environmental conditions may be lethal under other conditions. Scott *et al*. observed that over 90% of human cancer cells from a wide range of tumors and established cell lines died *in vitro* following arginine deprivation, while normal cells survived [27]. It is unlikely that all the susceptible cancer cells had mutations in genes involved in the synthesis of the NEAA arginine. Probably, arginine deprivation forced cells to activate a variety of genetic adaptation programs, which were functional in normal cells but not in cancer cells. The accumulation of DNA alterations in cancer cells during carcinogenesis probably inactivated the genetic programs required to adapt to and survive in the new environment created when arginine was deprived.

### Overcoming proteolysis by selective amino acid restriction

Restricting any AA *in vitro* is easy. One just has to prepare a medium without the desired AA and to add it to the cells. Restricting an AA *in vivo* is not that easy. The reason is that we have mechanisms for sensing and responding to AA deficiencies. Proteolysis is a key response mechanism to AA deprivation. Proteins are a source of AAs, and whole-body proteolysis and proteolysis at the cellular level can supply free AAs if their plasma or cellular levels are low. At the organism level, skeletal muscle proteolysis plays a key role in keeping adequate AA plasma concentrations during fasting periods. Liver proteolysis also plays a role. At the cellular level, protein breakdown during autophagy produces free AAs under conditions of AA limitation [28–31]. Some cancer cells, such as pancreatic cancer cells, are known to use macropinocytosis to transport extracellular proteins (e.g., albumin) into the cell. The internalized proteins undergo lysosomal degradation and produce free AAs [32,33]. This suggests that the dietary restriction of AAs will be buffered by the activation of proteolysis at the organism level and at the cellular level.

Although cells and organisms have mechanisms for sensing AA deficiencies, some of these mechanisms do not sense deficiencies in each of the 20 proteinogenic AAs. A sensing mechanism for each AA is not always necessary, mainly because they come together in the diet and because proteolysis provides all of them. During fasting, sensing one or several AAs may be sufficient to activate muscle proteolysis and elevate the levels of the 20 AAs. Evidence suggests that the levels of the EAA leucine may play a key role in controlling muscle protein metabolism; leucine supplementation stimulates muscle protein synthesis and reduces muscle protein breakdown even when the levels of other AAs are decreased [34, 35]. The levels of leucine required to inhibit muscle proteolysis seem to be higher than those for activating protein synthesis [36]. Leucine supplementation may therefore prevent muscle proteolysis during temporal restriction of specific AAs. Keeping an adequate cell volume in liver cells with sufficient levels of specific AAs, such as leucine and glutamine, may prevent liver proteolysis [28].

The mechanistic (or mammalian) target of rapamycin complex 1 (mTORC1) is a cellular nutrient sensor that plays a key role in the control of protein synthesis and degradation [30, 37]. mTORC1 activity strictly depends on sufficient intracellular AA levels. AA restriction leads to mTORC1 inhibition, which in turn results in autophagy activation, lysosomal degradation of cellular proteins, and generation of free AAs. However, mTORC1 is not equally sensitive to all AAs; leucine, arginine and glutamine have been identified as key activators of mTORC1 [30, 37, 38]. Leucine is particularly important for its activation. Evidence suggests that leucyl-tRNA synthetase senses increased leucine levels and activates mTORC1 in order to suppress autophagy [39]. Supplementation of leucine may sustain mTORC1 activity, thereby preventing autophagy-mediated proteolysis during temporal restriction of specific AAs. It has also been reported that glutamine activates the cellular uptake of leucine and can therefore facilitate leucine-induced mTORC1 activation and autophagy inhibition [40]. Supplementation of sufficient levels of glutamine and leucine may prevent the activation of autophagy during AA restriction.

The general AA control non-derepressible 2 (GCN2) kinase plays a key role in sensing deficits of any proteogenic AA [30,37]. Since no AA compensates for the absence of another during protein synthesis, GCN2 plays a key role in sensing low levels of each of the 20 proteogenic AAs. When an AA is scarce, its cognate aminoacyl transfer RNA synthetase fails to load the tRNA. The unloaded tRNA is detected by GCN2 kinase, which represses global protein synthesis by inhibiting the eukaryotic initiation factor 2α (eIF2α) kinase. At the same time, it activates the transcription of genes involved in the synthesis and cellular uptake of AAs in order to compensate the deficit. Although GCN2 allows for the detection of low levels of any proteinogenic AA in the context of an abundance of the other 19 AAs, it is important to realize that detecting the deficit is not sufficient to compensate it. The cell may need to activate genetic programs to obtain adequate levels of the restricted AA. These genetic programs may not be functional in cancer cells. In addition, the cells may need to move out of the cell cycle into a quiescent state until the deficit is overcome. Cancer cells may be unable to do so because of their DNA alterations.

Recent evidence supports a crosstalk between the GCN2-eIF2α and the mTORC1 signaling pathways to induce autophagy in response to nutrient deprivation [41]. This supports the possibility that GCN2 might detect restriction of any proteogenic AA and activate autophagy even in the presence of adequate levels of the rest of AAs. Even so, it is important to realize that cells cannot survive a prolonged restriction of any AA if they are unable to synthesize it or to obtain it from external sources. The continuous degradation of cellular components through autophagy will inevitably result in cell death.

Macropinocytosis of extracellular proteins in cancer cells may limit the efficacy of the anticancer strategy proposed in this manuscript. Macropinocytosis is a process in which extracellular fluid and its contents are internalized into cells through large vesicles known as macropinosomes. Some malignant cells, such as pancreatic cancer cells, can use macropinocytosis to transport extracellular proteins into the cell. The internalized proteins undergo lysosomal degradation and produce free AAs [32, 33]. This suggests that a selective AA restriction therapy (SAART) may be ineffective for cancer cells taking extracellular proteins through macropinocytosis. However, recent data indicate that the utilization of extracellular proteins as a source of AAs is suppressed by mTORC1 [42]. Since mTORC1 activity depends on adequate intracellular levels of particular AAs, supplementation of these AAs may sustain mTORC1 activity and prevent degradation of extracellular proteins. Alternatively, macropinocytosis can be selectively inhibited with Na^+^/H^+^ exchanger inhibitors such as amiloride (a diuretic drug) or 5-(N-Ethyl-N-isopropyl) amiloride [33,43].

## CONCLUDING REMARKS

The anticancer strategy proposed in this manuscript consists of treating cancer patients with a protein-free artificial diet in which the levels of particular AAs are manipulated. Some AAs are eliminated or restricted. Others are increased or kept unchanged in relation to their normal intakes. The aim is to create a challenging AA imbalance, which will force cells to activate genetic programs to obtain adequate levels of the 20 proteinogenic AAs. Normal cells can use their functional genome to adapt to and resist this temporal challenging environment. Cancer cells, however, may be unable to do so. Their extremely altered DNA may compromise their ability to activate the genetic programs required to survive the new environment.

*In vitro* data have already shown that AA restriction can kill a wide range of cancer cells without affecting normal cells. For example, cells from a variety of tumors and established lines died quickly *in vitro* following arginine deprivation [27]. When normal cells and cancer cells were grown together in arginine-free medium, the normal cells survived while the cancer cells died [27]. Depriving cells of particular AAs *in vivo* is challenging, because whole-body proteolysis can supply the AAs we restrict. However, experimental data indicate that proteolysis can be prevented when the levels of particular AAs are high. This suggests that we can create a challenging AA imbalance *in vivo* if we restrict specific AAs and keep high levels of others whose deficit triggers proteolysis. In fact, dietary deprivation of individual AAs for two weeks in mice resulted in significant alterations in the plasma levels of many proteinogenic AAs [44]. Restricting several AAs simultaneously, while increasing the levels of others, may lead to more marked AA imbalances.

It is difficult to predict at this time the most effective SAARTs, mainly because the mechanisms by which organisms and cells sense and respond to fluctuations in AA levels are far from been understood [30]. The good news is that there are only 20 proteinogenic AAs to manipulate. The possible combinations are finite, and their anticancer efficacy can be tested experimentally. Fully understanding how we sense and respond to AA restriction is not necessary to develop effective SAARTs. It would help, but it is not necessary.

Although none of the possible AA combinations should be ruled out, evidence suggests that some combinations may be more effective than others. Keeping high levels of leucine seems to be important to prevent whole-body and cellular proteolysis [30, 34, 35, 37–39]. Keeping sufficient levels of glutamine, a common fuel for cancer cells [45], may also be important to prevent proteolysis [28, 29, 40]. Restricting NEAAs may be more effective than restricting EAAs. Neither normal cells nor cancer cells can synthesize EAAs. However, normal cells can synthesize NEAAs while cancer cells are probably unable to obtain all of them. This difference may confer selectivity. The idea is not to find the most toxic combination for cancer cells, but the most selective. Restricting several AAs together may be more effective than restricting AAs individually. Because the presence of some AAs can compensate for the deficit of others, it may be important to restrict complementary AAs simultaneously. For example, serine is required for the synthesis of cysteine, and serine and glycine are interconvertible through the enzymes serine hydroxymethyltransferases SHMT1 and SHMT2 (Figure 1). Restricting these three NEAAs together may force cells to activate a variety of genetic programs, some of which may be inactivated in cancer cells. In fact, *TP53* gene (which encodes p53 protein) is the most frequently mutated gene in cancer, and evidence suggests that p53- defficient tumors are vulnerable to serine starvation [46]. Rapidly proliferating cancer cells from a variety of tissues, but not rapidly proliferating normal cells, are also vulnerable to glycine deprivation [47]. Restricting cysteine may also be important to reduce the synthesis of the tripeptide glutathione (γ-L-glutamyl-L-cysteinylglycine); cancer cells may need high levels of the antioxidant glutathione to cope with the oxidative stress resulting from serine deprivation [46]. Reducing the levels of the EAA methionine (a precursor of cysteine) may increase the toxicity of this combination to cancer cells, but perhaps to normal cells too. Alternatively, if the restriction of serine, glycine and cysteine is enough to kill the cancer cells, increasing the levels of methionine may reduce the toxicity of this combination towards normal cells and make it more selective. Again, the key is not to find the most toxic combination for cancer cells, but to find a combination able to eliminate the cancer cells without significantly affecting our normal body cells.

Properly testing anticancer potential *in vitro* requires using cancer cells and a variety of nonmalignant cells [2, 48, 49]. The experimental approach used by Scott *et al.* [27] is adequate to detect the *in vitro* anticancer potential of any AA combination. However, restricting rather than depriving AAs may be more translatable to an *in vivo* situation. The information obtained *in vitro* will be valuable, but limited. Whole-body proteolysis is the main barrier to any SAART, and this parameter cannot be studied *in vitro*.

*In vivo* experiments may be necessary to properly screen the possible SAARTs. The following experimental approach could be used to screen these potential therapies easily, rapidly and reliably. First, inject cancer cells (e.g., mouse B16F10 melanoma cells) into the tail vein of 2-3 mice (e.g., normal C57BL/6 mice) per group, and wait 1-2 weeks so that lung metastases are fully established. Second, change their normal diet for a protein-free artificial diet in which the levels of particular AAs are manipulated; after a few days or weeks, change the artificial diet for the normal diet. Third, evaluate survival as an endpoint for efficacy assessment, that is, wait a few days or weeks to evaluate if the mice treated with the artificial diet live longer than the untreated mice. In the original protocols, the animals are euthanized 12-20 days after the tail vein injection, and each mouse usually has 200-300 pulmonary metastases when injected with 5 × 10^4^ cells from a highly metastatic cell line (e.g., B16F10 melanoma cells) [50,51]. To my knowledge, current anticancer therapies cannot save the lives of these mice when treatments are started once the metastases are fully established. Untreated mice and mice treated with ineffective SAARTs will die quickly; results can be obtained fast. Effective treatments should be confirmed using more mice. The efficacy of a treatment should also be confirmed in additional metastatic models (e.g., metastatic xenograft models) using different types of cancer cells. Using human cancer cells from a variety of tissues and with different DNA alterations will help predict what cancer types are susceptible to a particular SAART. One should always have in mind that an experimental therapy should improve the survival of the standard treatment before advancing into clinical testing [49]. Any research team with cell culture and animal facilities can easily conduct these experiments. Unfortunately, my team does not have funds to carry them out.

When designing and testing possible SAARTs, it is essential to provide adequate nitrogen levels so that normal cells can synthesize the restricted NEAAs. A nitrogen-deficient diet will probably trigger whole-body proteolysis. So, when we reduce the levels of some AAs, we should increase the levels of others or provide an alternative nitrogen source. Most proteinogenic AAs produce glutamate during their degradation, and glutamate provides the amino group for the synthesis of most NEAAs (Figure 1). This should facilitate the design of nitrogen-balanced diets. It is also important to keep adequate levels of other nutrients, such as glucose and fatty acids, by supplying sufficient quantities of carbohydrates and fats. Deficits in these nutrients will probably trigger proteolysis. For example, hypoglycemia increases glucagon levels and triggers whole-body proteolysis, while hyperglycemia increases insulin levels and counteracts the proteolytic effect of glucagon [31, 52, 53]. If the artificial diet is hypocaloric, the cellular ATP:AMP ratios may decrease; this will activate autophagy even in the presence of adequate levels of AAs [30]. It should also be noted that animals and patients may reject artificial diets lacking particular AAs, especially diets lacking EAAs [54]. Cells in the brain's anterior piriform cortex can sense AA deficiencies through the GCN2 kinase and signal food rejection [54]. Ensuring a sufficient intake of the artificial diet is important, because we need to keep high levels of the nutrients that prevent proteolysis.

The plasma half-life of the nutrients (or drugs) used to prevent proteolysis needs to be carefully considered to design the best administration protocols. For example, the increased levels of leucine achieved in blood after its oral administration do not last long [55]. This means that soon after the ingestion of an artificial diet rich in leucine, the body levels of this EAA may be insufficient of prevent whole-body proteolysis. The activation of proteolysis will increase the levels of the AAs we are restricting and will limit SAART efficacy. This limitation can be overcome by continuous feeding. In patients, this can be accomplished by continuous nasogastric feeding, or with a continuous intravenous infusion of an equivalent parenteral diet. Nasogastric feeding will probably result in higher concentrations of AAs in the liver, because nutrients absorbed from the gastrointestinal tract are delivered to the liver by the portal vein before reaching the general circulation (hepatic first-pass effect). Keeping high concentrations of AAs in the liver may be important to prevent liver proteolysis. Hepatic enzymes, however, may produce some of the restricted AAs and deliver them to the general circulation.

Although SAART may be effective as a stand-alone anticancer therapy, it may be necessary to combine it with drugs. If keeping high levels of particular AAs is not enough to prevent proteolysis sufficiently, insulin may be required to increase SAART efficacy. Insulin prevents whole-body proteolysis, especially muscle proteolysis [31]. Insulin also facilitates the cellular uptake of glucose and the activation of glycolysis. Because glycolysis provides ATP, this effect of insulin may be important to keep adequate cellular ATP:AMP ratios and thus avoid autophagy. In addition, glucose uptake and glycolysis are necessary to provide building blocks for the synthesis of NEAAs (Figure 1). Inhibitors of the Na^+^/H^+^- exchanger may be needed to prevent macropinocytosis of extracellular proteins in some cancers [32, 33, 43]. Because sodium ions play a key role in AA transport across cell membranes [56], the use of Na^+^/H^+^-exchanger inhibitors (e.g., amiloride) and Na^+^/K^+^-ATPase inhibitors (e.g., cardiac glycosides) can alter AA transport across cell membranes and might help create an AA imbalance.

SAART may also be combined with standard anticancer treatments. For example, high levels of the tripeptide glutathione (GSH) confer resistance to a wide range of anticancer drugs [57–59], including the commonly used anticancer agent cisplatin [60]. Inhibitors of GSH synthesis and of GSH-dependent detoxifying enzymes have been developed [58,59]. These inhibitors increase the toxicity of many anticancer agents to cancer cells. However, these combinations induce toxicity to normal cells too. The reason is that normal cells also need GSH and GSH-dependent enzymes to protect themselves against these drugs and against the reactive oxygen species (e.g., hydrogen peroxide) produced during normal cell metabolism. As discussed before, restriction of the NEAAs cysteine, glycine and serine may compromise the synthesis of GSH in cancer cells, but not in normal cells. Normal cells would use GSH to detoxify the anticancer drugs and would survive. Cancer cells may be unable to do so and would die. Treatment of cancer patients with an adequate SAART (e.g., Cys-, Gly-, Ser-, Leu+, Gln+?, insulin+?) may selectively inhibit GSH synthesis in cancer cells. This may increase the selectivity of anticancer drugs such as cisplatin, which would result in improvements in the survival of cancer patients.

It is becoming widely accepted that each cancer type, and even each cancer patient, may require a different therapy. The extensive mutational heterogeneity observed between and within tumors supports this view [17, 61]. Evidence discussed in this manuscript indicates, however, that SAART may be effective against all types of cancer cells. All cells need to synthesize proteins, and all cancer cells have DNA alterations that may compromise their ability to obtain adequate levels of the 20 AAs required for protein synthesis. In addition, experimental and theoretical evidence suggests that specific SAARTs may be effective not only against all the cancer cells within a tumor, but also against a variety of tumor types. Experimental observations have revealed that every cancer cell within a tumor often contains the same core set of genetic alterations, with heterogeneity confined to mutations that emerge late during tumor growth [61, 62]. The stem cell division theory of cancer [5–7] can explain these experimental observations. If cancer arises from normal stem cells, all the mutations occurring in these cells before becoming malignant (CSCs) will be found in all their progeny, that is, in all the tumor cancer cells. Obviously, some tumor cells may lack some of these mutations if they lose during cell division the chromosomes or pieces of chromosomes that bear these DNA alterations. The mutations arising during the self-renewal of CSCs will be found only in the tumor populations derived from these malignant stem cells. In addition to self-renewing, CSCs generate progenitor cancer cells, which divide and produce the bulk of cancer cells within a tumor. The mutations found in few tumor cancer cells probably occur during the division of these progenitor cells. In some cases, the tumor cancer cells may arise from more than one normal stem cell. In these cases, not all the cancer cells within a tumor will share the same core set of genetic alterations. In short, experimental and theoretical evidence indicates that all the tumor cancer cells share the same core set of DNA alterations in most cases; therefore, all the tumor cells within a tumor may be vulnerable to the same SAART. Experimental data also suggest that different tumor types may be vulnerable to the same SAART. As discussed before, restriction of just one AA (i.e., arginine, serine or glycine) may be sufficient to kill many cancer cells of different tissues and genetic backgrounds [27, 46, 47]. Patients with different tumor types may therefore respond well to the same SAARTs. Naturally, this does not mean that all cancer patients will respond to the same SAART, or that all the cancer cells within a tumor will always respond to the same SAART. Sequencing different SAARTs should be considered when this occurs or to prevent this from happening.

SAART may also be used to prevent cancer, especially in people at high risk of developing the disease (e.g., aged people). SAART may be an effective strategy to selectively kill mutated stem cells before they give rise to cancer. Some mutations occurring early in carcinogenesis may confer sensitivity to particular SAARTs. For example, stem cells that develop mutations in the *TP53* tumor suppressor gene may be vulnerable to serine restriction [46]. Although SAART may be a valuable preventive strategy, one should always keep in mind that the restriction of specific AAs (especially EAAs) may be highly toxic if proteolysis is inhibited. The possible toxicity associated with particular SAARTs should be carefully considered if they are to be used in healthy people.

Because all proteinogenic AAs are necessary for cell survival, our body should always provide an adequate supply of all of them if their dietary intake is low. However, evidence discussed in this manuscript indicates that our body will not sufficiently respond to deficits in specific AAs if the levels of other AAs and nutrients (e.g., glucose) are adequate. If proteolysis is sufficiently blocked by the presence of adequate levels of particular nutrients, a diet lacking specific proteinogenic AAs may cause cytotoxicity and may be fatal. This should not happen because our muscles store large quantities of all proteinogenic AAs. This possible biological flaw may be the key to successfully treating cancer.

## SUPPLEMENTARY FIGURE


