# Supplementary material for: Selective amino acid restriction therapy (SAART): a non-pharmacological strategy against all types of cancer cells
Source: Oncoscience. 2015 Nov 10;2(10):857–66. doi: 10.18632/oncoscience.258 (PMC4671952; doi:10.18632/oncoscience.258)
Supplement: Supplementary file 1 [file oncoscience-02-0857-s001.pdf]

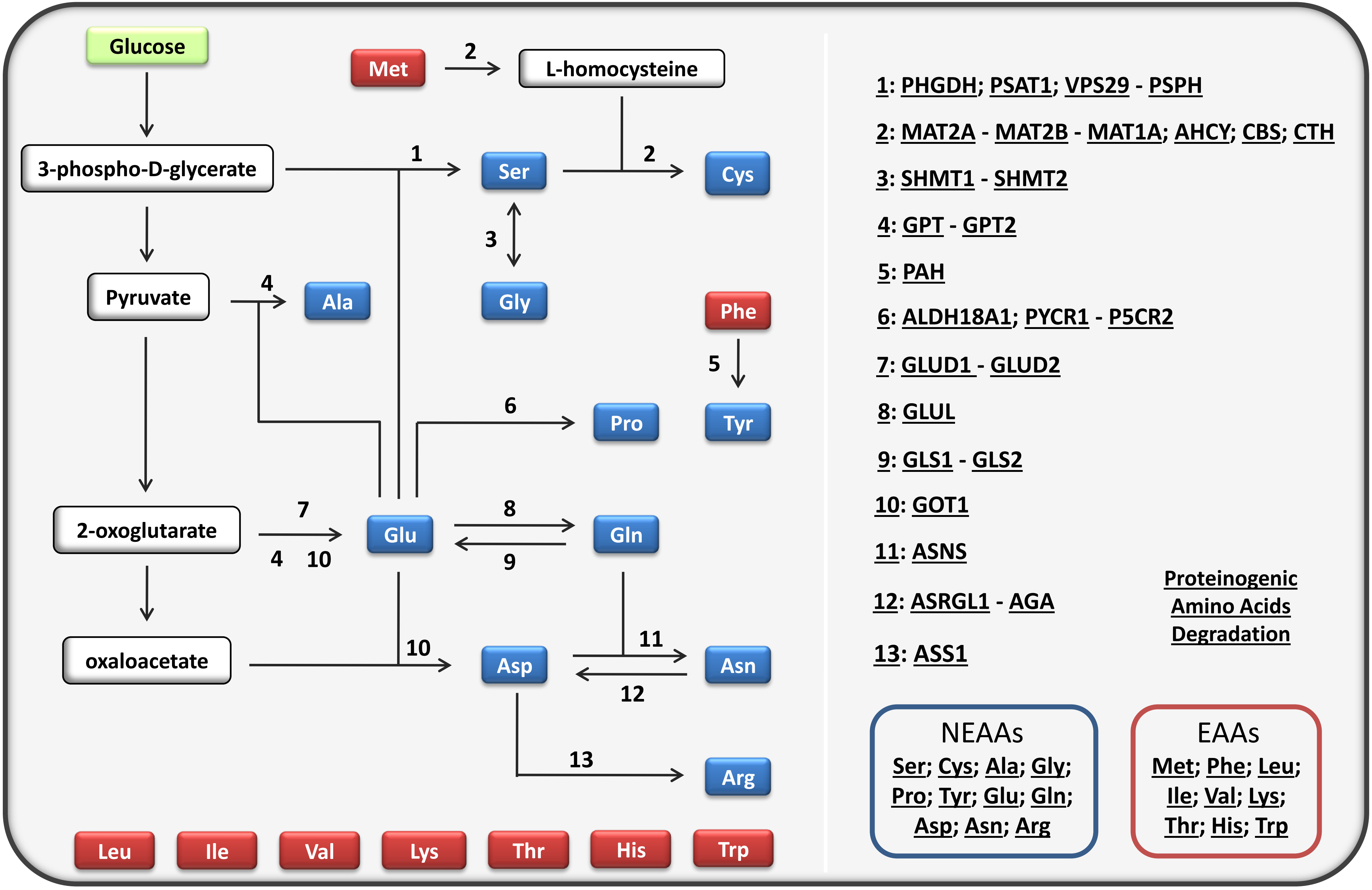

Glucose
Met
2
L-homocysteine
3-phospho-D-glycerate
1
Ser
2
Cys
3
Pyruvate
4
Ala
Gly
Phe
5
6
Pro
Tyr
7
2-oxoglutarate
4
10
Glu
8
9
Gln
oxaloacetate
10
Asp
11
12
Asn
13
Arg
Leu
Ile
Val
Lys
Thr
His
Trp
1: PHGDH; PSAT1; VPS29 - PSPH
2: MAT2A - MAT2B - MAT1A; AHCY; CBS; CTH
3: SHMT1 - SHMT2
4: GPT - GPT2
5: PAH
6: ALDH18A1; PYCR1 - P5CR2
7: GLUD1 - GLUD2
8: GLUL
9: GLS1 - GLS2
10: GOT1
11: ASNS
12: ASRGL1 - AGA
13: ASS1
Proteinogenic Amino Acids Degradation
NEAAs
Ser; Cys; Ala; Gly; Pro; Tyr; Glu; Gln; Asp; Asn; Arg
EAAs
Met; Phe; Leu; Ile; Val; Lys; Thr; His; Trp
